# Activated IL-1RI Signaling Pathway Induces Th17 Cell Differentiation *via* Interferon Regulatory Factor 4 Signaling in Patients with Relapsing-Remitting Multiple Sclerosis

**DOI:** 10.3389/fimmu.2016.00543

**Published:** 2016-11-29

**Authors:** Yonggang Sha, Silva Markovic-Plese

**Affiliations:** ^1^Department of Neurology, University of North Carolina at Chapel Hill, Chapel Hill, NC, USA; ^2^Department of Microbiology and Immunology, University of North Carolina at Chapel Hill, Chapel Hill, NC, USA

**Keywords:** IL-1, IL-1R, IRF4, Th17 cells, multiple sclerosis

## Abstract

IL-1β plays a crucial role in the differentiation of human Th17 cells. We report here that IL-1RI expression is significantly increased in both naive and memory CD4^+^ T cells derived from relapsing-remitting multiple sclerosis (RR MS) patients in comparison to healthy controls. Interleukin 1 receptor (IL-1R)I expression is upregulated in the *in vitro*-differentiated Th17 cells from RR MS patients in comparison to the Th1 and Th2 cell subsets, indicating the role of IL-1R signaling in the Th17 cell differentiation in RR MS. When IL-1RI gene expression was silenced using siRNA, human naive CD4^+^ T cells cultured in the presence of Th17-polarizing cytokines had a significantly decreased expression of interleukin regulatory factor 4 (IRF4), RORc, IL-17A, IL-17F, IL-21, IL-22, and IL-23R genes, confirming that IL-1RI signaling induces Th17 cell differentiation. Since IL-1R gene expression silencing inhibited IRF4 expression and Th17 differentiation, and IRF4 gene expression silencing inhibited Th17 cell differentiation, our results indicate that IL-1RI induces human Th17 cell differentiation in an IRF4-dependant manner. Our study has identified that IL-1RI-mediated signaling pathway is constitutively activated, leading to an increased Th17 cell differentiation in IRF4-dependent manner in patients with RR MS.

## Introduction

IL-1β induces differentiation of T helper (Th) 17 cells, which play a critical role in the development of the autoimmune response. IL-1β contributes to IL-6-induced RORc expression and IL-17A production in humans (1, 2), consistent with animal studies in which IL-1β has a synergistic effect with IL-23 in promoting Th17 differentiation (3, 4). The role of IL-17 in MS and other autoimmune disorders has been demonstrated in multiple studies. IL-17 mRNA and protein expression is elevated in the active MS brain lesions (5, 6), where it is present in both CD4^+^ and CD8^+^ T cells, as well as in astrocytes and oligodendrocytes. IL-17 gene expression is significantly higher in mononuclear cells derived from the blood and cerebrospinal fluid (CSF) of MS patients when compared to healthy controls (HCs), and the numbers of IL-17-expressing blood mononuclear cells are higher during MS exacerbations in comparison to clinically silent periods (7). IL-17 has also been detected in the target organs of other autoimmune diseases, including rheumatoid arthritis, psoriasis, and autoimmune uveitis (8), suggesting its role in human autoimmune responses.

Upon binding of IL-1 to IL-1RI, an interleukin-1 receptor (IL-1R) accessory protein (AcP) is recruited to form a high affinity IL-1RI-IL-1RAcP heterodimeric receptor, which initiates the recruitment of the MyD88 adapter and its activation and association with IRAK4. Tumor necrosis factor (TNF) receptor-associated factor (TRAF) 6 is subsequently recruited to the receptor-associated IRAK1–IRAK4–MyD88 adaptor protein complex. The formation of this complex leads to the induction of the MAPK p38 cascade and the activation of NF-κB signaling pathway (9–11). These signaling pathways lead to the expression of a range of cytokines involved in inflammatory responses.

Interleukin-1 receptor signaling in T cells is required for the early programing of Th17 cell differentiation and Th17 cell-mediated autoimmunity (12). Th17 cells, which are characterized by the production of IL-17, are considered a key player in the pathogenesis of autoimmune diseases (6, 13). Naive CD4^+^ T cells require IL-1β plus IL-23, IL-6, and TGFβ to differentiate into Th17 cells (1, 14–17). Transcription factors STAT3, RORα and RORγτ determine Th17 cell lineage-specific differentiation program through induction of a set of signature cytokines and cytokine receptors, including IL-1R (18, 19). IL-1R1 expression in T cells is necessary for the Th17 cell differentiation (12, 20). Mice defective in IL-1RI signaling were resistant to experimental allergic encephalomyelitis (EAE) and exhibit a defect in the generation of IL-17-producing T cells, suggesting that IL-1RI signaling in T cells is required for Th17 cell differentiation (21, 22). Single immunoglobulin IL-1-related receptor (SIGIRR), a negative regulator of IL-1R signaling, was induced during Th17 cell commitment and suppressed Th17 cell proliferation through its inhibitory effects on IL-1R signaling (22). In human peripheral blood, CD4^+^ T cells that express IL-1RI have the capacity to produce IL-17 in naive and memory T cells. IL-R1^+^ memory cells had increased gene expression of IL-17, RORc, and interleukin regulatory factor 4 (IRF4), even before T cell receptor (TCR) triggering, suggesting that IL-1R1 expression renders cells committed to Th17 differentiation (20). IL-1RI^+^ naive CD4^+^ T cells produce higher levels of IL-17 in response to a combination of IL-1β and TCR triggering compared to IL-1RI^−^ naive CD4^+^ T cells, which are capable of producing IL-17 only upon TCR stimulation-induced IL-1R1 expression (20).

Previous studies have shown that the human Th17-polarizing cytokines are different from those in mice. In mice, IL-6 plays a critical role and IL-1β exerts an enhancing effect, whereas in humans, IL-1β is essential and IL-6 enhances its effect on Th17 cell differentiation. It has been reported that, in healthy individuals, IL-1β alone induces RORc and IL-17 expression, which is sustained in the presence of IL-6. Moreover, in cultures stimulated with IL-1β or IL-1β plus IL-6, the addition of IL-23 increases the percentage of IL-17-producing T cells in long-term cultures, which is more prominent in cultures stimulated with IL-1β alone (20).

The goal of this study was to characterize the role of IL-1R1 signaling in promoting human Th17 cell differentiation in the context of their role in the development of the autoimmune response in patients with relapsing-remitting multiple sclerosis (RR MS). Our results may provide rationale for therapeutic targeting of IL-1RI in autoimmune diseases.

## Materials and Methods

### Study Subjects

Thirty-two patients with confirmed diagnoses of RR MS and 35 HCs were enrolled in the study upon signing an institutional review board (IRB)-approved informed consent. The study was approved by the University of North Carolina IRB. The inclusion criteria for the MS patients consisted of a confirmed diagnosis of RR MS according to McDonald’s diagnostic criteria, age 18–55, and an extended disability status score (EDSS) 1.5–5.5. The patients were not treated with immunomodulatory therapy at the time of blood collection. The treatment-free period was at least 6 weeks for IV methylprednisolone, 3 months for interferon-beta and glatiramer acetate, and 6 months for tecfidera, teriflunomide, and fingolimod.

### Cell Cultures

CD4^+^, CD4^+^CD45RA^+^, and CD4^+^CD45RO^+^ T cells were isolated from peripheral blood mononuclear cells (PBMCs) using magnetic beads (Miltenyi Biotech). Cell purity was confirmed by flow cytometry (>95%). Cells were maintained after separation in RPMI 1640 complete medium supplemented with 10% fetal bovine serum (FBS), 2 mM glutamine, and 100 U/ml penicillin G and streptomycin. For the *ex vivo* gene expression measurements, CD4^+^, CD4^+^CD45RA^+^, and CD4^+^CD45RO^+^ T cells were cultured at 2 × 10^6^ cells/ml in serum-free medium (Opti-MEM I, Gibco) for 6 h prior to RNA extraction. In order to generate different T cell subsets, CD4^+^CD45RA^+^ naive T cells were stimulated with plate-immobilized anti-CD3 (1 μg/ml) and anti-CD28 (5 μg/ml) mAb and cultured in serum-free medium in the absence or presence of (a) Th1-polarizing cytokines IL-12 (10 ng/ml), IFN-γ (3 ng/ml), and anti-IL-4 mAb (10 ng/ml); (b) Th2-polarizing cytokines IL-4 and anti-IL-12 mAb (10 μg/ml); or (c) Th17-polarizing cytokines IL-1β, IL-6, IL-23 (all at 50 ng/ml), TGFβ (10 ng/ml), anti-IL-4 mAb, anti-IL-27 mAb, and anti-IFNγ mAb (all at 10 μg/ml). After 72 h, the cells were harvested for gene expression RT-PCR studies. In the Th17 cell differentiation experiments following IL-11R and IRF4 siRNA, the cells were harvested at 72 h for RNA extraction, and the supernatants (SNs) were collected from the same cell cultures for cytokine measurement by ELISA.

### Quantitative RT-PCR

Total RNA was isolated from CD4^+^, CD4^+^CD45RA^+^, CD4^+^CD45RO^+^ T cells and reverse-transcribed to cDNA using an iSCRIPT cDNA synthesis kit (Bio-Rad). Quantitative RT-PCR (qRT-PCR) was performed using an Applied Biosystems PRISM 7700 Sequence System. The primers were purchased from Applied Biosystems. Each sample was analyzed in triplicate. Relative gene expression was expressed upon normalization against 18S RNA.

### siRNA Experiment

The siRNAs for IL-1R1, IRF4, and control A siRNA were purchased from Santa Cruz Biotechnology. 2 × 10^6^ CD4^+^CD45RA^+^ cells per condition were transfected with each of the listed siRNAs using human T cell Nucleofector kit (Lonza). They were then stimulated with plate-immobilized anti-CD3 (1 μg/ml) and anti-CD28 (5 μg/ml) mAb and cultured in serum-free medium (Opti-MEM I, Gibco) in the absence or presence of Th17-polarizing cytokines. After 72 h, the cells were harvested for gene expression studies and their cytokine production was measured in SNs.

### Western Blotting

CD4^+^CD45RA^+^ cells were plated at 2 × 10^6^ cells per condition for Western blotting. The cells were lysed with lysis buffer containing 2.5 mM sodium pyrophosphate, 1 mM NA3VO4, and 1 mM phenylmethylsulfonyl fluoride (Santa Cruz Biotechnology). The cell lysates were resolved with 5–15% gradient SDS-PAGE (Bio-Rad) and transferred to polyvinylidene difluoride (PVDF) membranes. The membranes were blocked with 5% milk in TBS (20 mM Tris and 500 mM NaCl) and 0.1% Tween 20 at room temperature for 1 h followed by overnight incubation at 4°C with primary Abs against IL-1RI (Abcam), IRF4 (Santa Cruz Biotechnology), RORc (Abcam), β-actin (Sigma-Aldrich). Secondary HRP-conjugated Ab (Santa Cruz Biotechnology) was added at a dilution of 1/2000 for 1 h and the protein bands were detected with an ECL Detection System (Santa Cruz Biotechnology).

### ELISA

Supernatants from the cell cultures were collected and stored at −80°C until the cytokine measurements. IL-17A, IL-17F, IL-21, IL-22 (all from eBiosciences), IL-4, and IFN-γ (both from BD Pharmingen) were measured in duplicate by ELISA following the manufacturer’s recommendation. Results are expressed for each subject as cytokine concentration in pictogram per milliliter.

### Statistics

Statistical analyses of the *ex vivo* qRT-PCR results were performed using a *t*-test analysis (GraphPad, InStat). Statistical analyses of the comparisons for multiple groups were performed using a repeated measure ANOVA (GraphPad, InStat). *p* Values <0.05 were considered significant.

## Results

### IL-RI Gene Expression Is Significantly Increased in Both Naive and Memory CD4^+^ Cells Derived from RR MS Patients in Comparison to HCs

Our previously published gene expression profiling study of the *ex vivo* separated PBMCs from patients with clinically isolated syndrome (CIS) suggestive of MS has reported that IL-1R is the most significantly upregulated gene in comparison to HCs (23).

**Figure 1:**
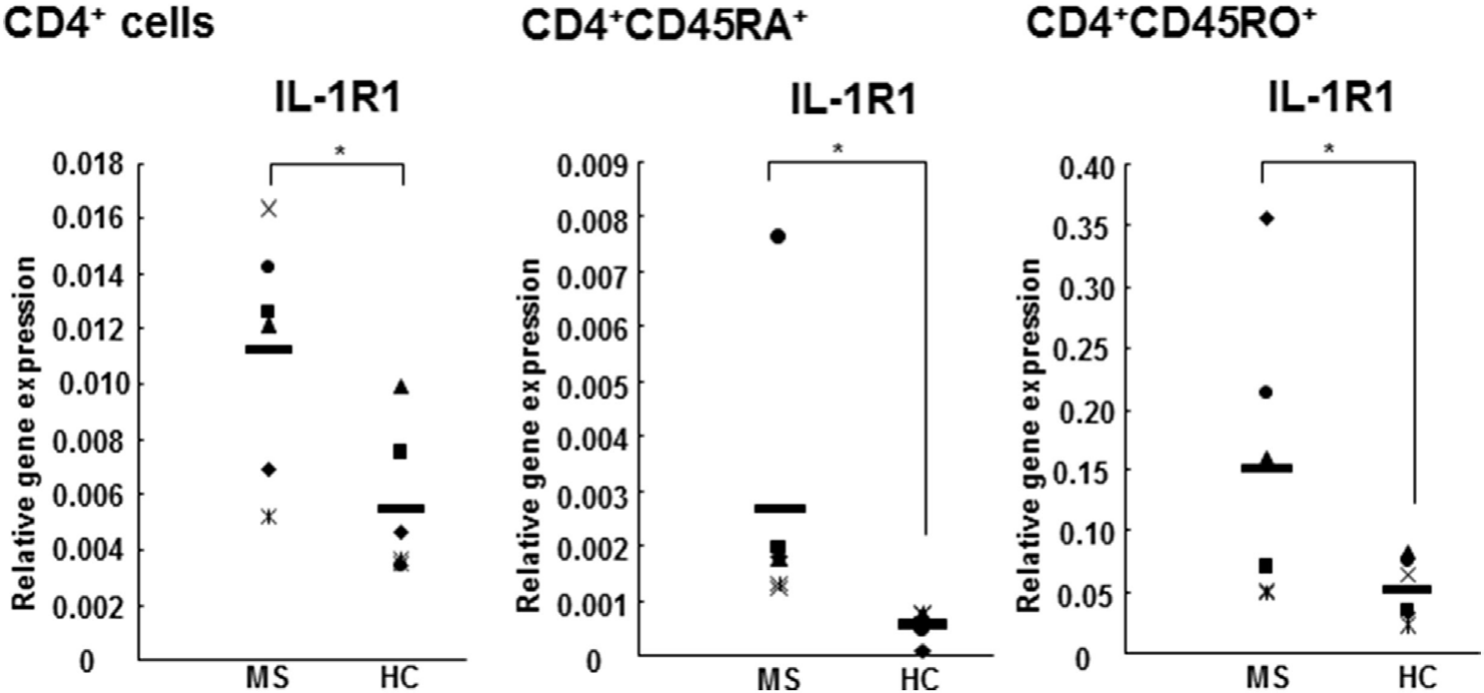
**IL-1RI gene expression is increased in CD4^+^, CD4^+^CD45RA^+^, and CD4^+^CD45RO^+^ cells from RR MS patients in comparison to HCs**. CD4^+^, CD4^+^CD45RA^+^ naive, and CD4^+^CD45RO^+^ memory T cells derived from six RR MS patients and six HCs were separated using magnetic beads. The total RNA was harvested and the gene expression of IL-1RI and 18S was measured using qRT-PCR. The results are presented as the relative gene expression normalized against 18S mRNA. Statistical analysis was performed using unpaired *t*-test.

In this study, we found an increased IL-1RI gene expression in CD4^+^, CD4^+^CD45RA^+^, and CD4^+^CD45RO^+^ cells derived from RR MS patients in comparison to HCs (Figure 1). The expression of IL-1RI on memory CD4^+^ cells is significantly higher than on the naive cells in both RR MS patient and HCs (Figure 1), consistent with the results in healthy individuals reported by Lee et al. (20). While those authors have studied the IL-1RI^+^ cells, which represent 20% of CD4^+^ cells in healthy donors, and identified that the frequency of naive CD4^+^ cells is lower, while the frequency of memory CD4^+^ cells is higher in IL-1RI^+^ than in IL-1RI^−^ cells, our results provide further insight into the differential gene expression of IL-1RI in both CD4^+^ cell subsets in RR MS patients in comparison to HCs.

### *In Vitro*-Differentiated Th17 Cells Express Significantly Higher Levels of IL-1RI in Comparison to Th1 and Th2 Cells

**Figure 2:**
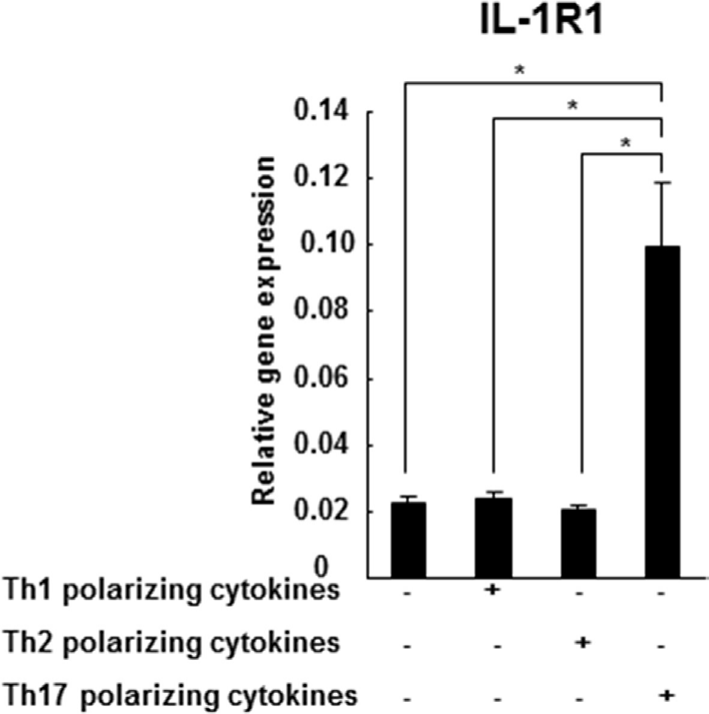
**IL-1R1 expression is higher in the *in vitro*-differentiated Th17 cells in comparison to Th1 and Th2 cultures**. CD4^+^CD45RA^+^ cells were derived from three HCs and stimulated with plate-immobilized anti-CD3 and anti-CD28 mAb in a serum-free medium in the absence or presence of Th1, Th2, or Th17-polarizing cytokines. After 72 h, total RNA was extracted and gene expression of IL-1R1 and 18S was measured by RT-PCR. The results are expressed as relative gene expression normalized for 18S mRNA expression. Statistical analysis was performed using ANOVA. ***p < 0.001.

IL-1RI gene expression was detected in naive CD4^+^CD45RA^+^ cells differentiated in the presence of Th1 (IL-12, IFN-γ, and anti-IL-4 mAb), Th2 (IL-4 and anti-IL-12 mAb), or Th-17 (IL-1β, IL-6, IL-23, TGF-β, anti-IL-4 mAb, anti-IL-27, and anti-IFN-γ mAb)-polarizing cytokines. We have identified that the *in vitro*-differentiated Th17 cells express significantly higher levels of IL-RI in comparison to the Th1 and Th2 cells (Figure 2). Our results are consistent with a previous study on the increased expression of IL1-RI in *in vitro*-differentiated Th17 mouse cells (12), as well as with our previously published study reporting higher IL-1R protein expression in IL-17A^+^CD4^+^ (Th17) cells in comparison to IFN-γ^+^CD4^+^ (Th1) and IL-4^+^CD4^+^ (Th2) cells from CIS patients (23).

### IL-1RI Activation Induces Human Th17 Cell Differentiation

**Figure 3:**
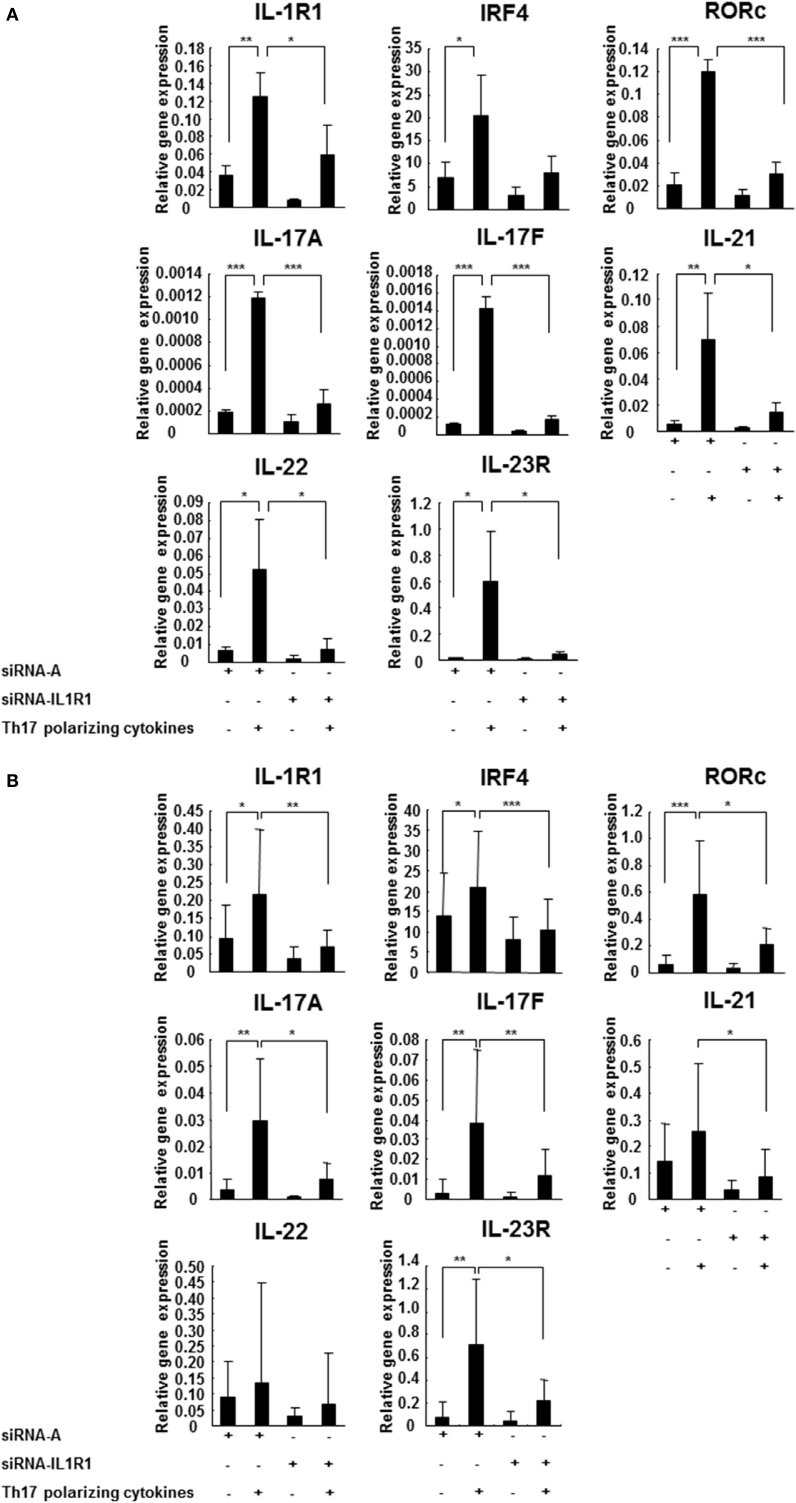
**IL-1R1 signaling induces Th17 cell differentiation**. CD4^+^CD45RA^+^ cells were derived from three HCs **(A)** and seven RR MS patients **(B)** and transfected with a control siRNA A or siRNA IL-1R1, stimulated with plate-immobilized anti-CD3 and anti-CD28 mAb and cultured in serum-free medium in the absence or presence of Th17-polarizing cytokines. The total RNA was extracted at 72 h, and the expression of the indicated genes was measured using RT-PCR. The results are expressed as relative gene expression normalized against 18S mRNA expression. Statistical analysis was performed using repeated measures ANOVA, *p < 0.05, **p < 0.01, ***p < 0.001.

In order to directly demonstrate the effect of IL-1RI signaling on the Th17 cell differentiation, control siRNA A, and siRNA IL-1RI-transfected naive CD4^+^ cells derived from HCs and RR MS patients were cultured in the absence or presence of Th17-polarizing cytokines. In the CD4^+^CD45RA^+^ cells derived from HCs, the Th17-polarizing cytokines induced a significantly higher expression of IL-1R1, IRF4, RORc, IL-17A, IL-17F, IL-21, IL-22, and IL-23R Th17 cell markers in comparison to baseline. In contrast, siRNA IL-1RI-transfected cells derived from HCs exhibited a decreased expression of those Th17-related genes upon Th17 differentiation (Figure 3A). In CD4^+^CD45RA^+^ cells derived from RR MS patients (Figure 3B), gene expressions of IL-1RI, IRF4, RORc, IL-17A, IL-17F, IL-21, and IL-23R were even more increased upon stimulation with Th17-polarizing cytokines than in HCs. The induction of IL-1RI, IRF4, RORc, IL-17A, IL-17F, IL-21, and IL-23R were inhibited in the siRNA IL-1RI-transfected cells. The studies of the naive CD4 cells with silenced IL-1RI expression confirmed that IL-1RI signaling is essential for human Th17 cell differentiation. Our results demonstrated preferential Th17 cell differentiation in RR MS patients in comparison to HCs, since the gene expression of IL-1R1, IRF4, RORc, IL-17A, IL-17F, IL-21, IL-22, and IL-23R were higher in MS patients than in HCs at baseline and following Th17 cell differentiation (Figures 3A,B).

### IL-1RI Signaling Is Required for the Secretion of Th17 Cytokines

**Figure 4:**
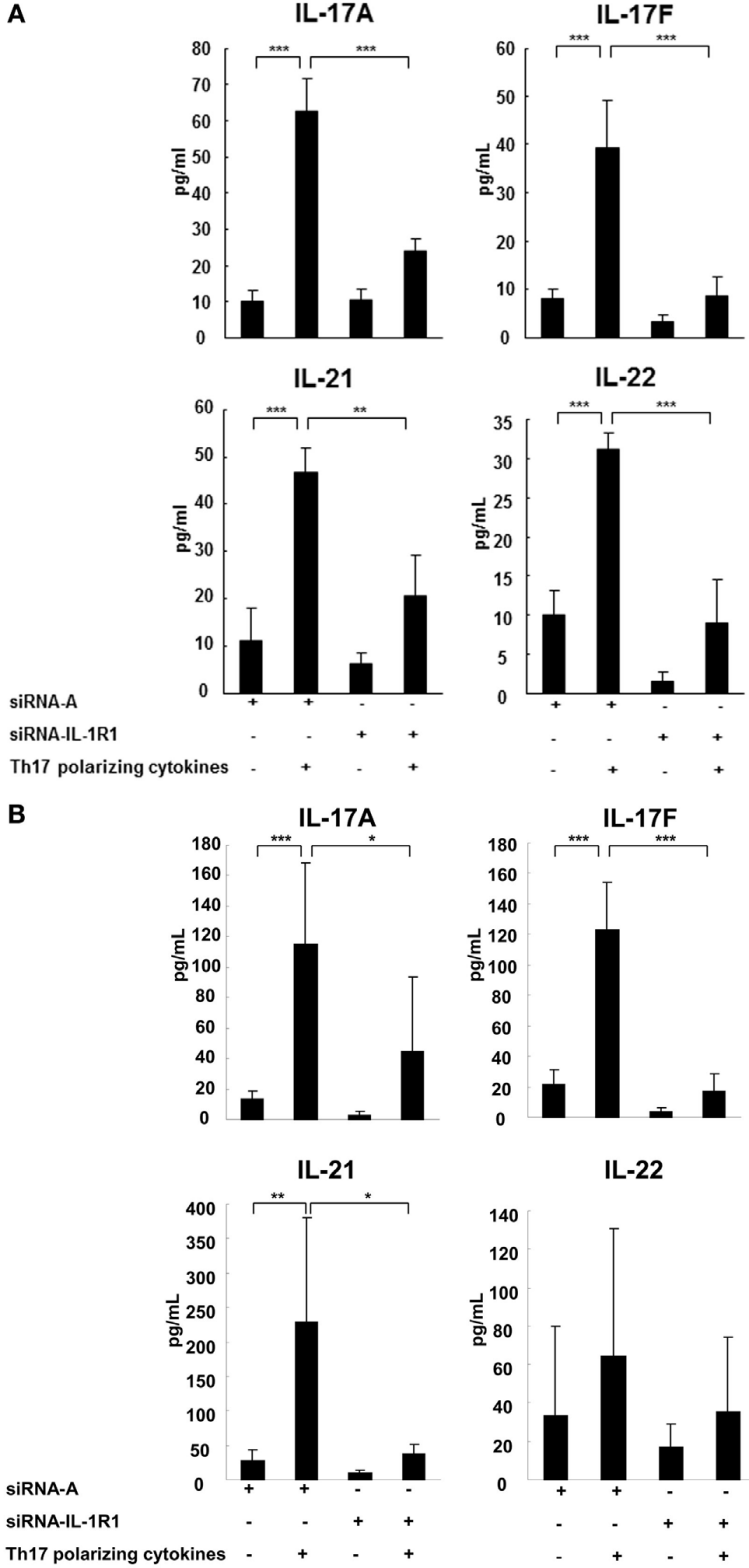
**IL-1R1 signaling induces secretion of Th17 cytokines**. CD4^+^CD45RA^+^ cells were derived from three HCs **(A)** and three RR MS patients **(B)**, transfected with a control siRNA A or siRNA IL-1R1, stimulated with plate-immobilized anti-CD3 and anti-CD28 mAb and cultured in serum-free medium in the absence or presence of Th17-polarizing cytokines. After 72 h, the supernatants were collected and the cytokine production measured by ELISA. Statistical analysis was performed using a repeated measures ANOVA, *p < 0.05, **p < 0.01, ***p < 0.001.

The subsequent studies have examined to what extent have IL-1RI-mediated signaling modified naive CD4^+^ cell cytokine secretion following Th17 cell differentiation. We measured cytokine concentrations in the SNs of the naive CD4^+^ cells with silenced IL-1RI expression in the absence or presence of Th17-polarizing cytokines using ELISA. The results revealed that IL-17A, IL-17F, IL-21, and IL-22 secretion induced in naive CD4^+^ cells derived from the HCs in the presence of Th17-polarizing cytokines were significantly suppressed upon silencing of their IL-1RI expression (Figure 4A). In the naive CD4^+^ cells derived from RR MS patients, IL-17A, IL-17F, and IL-21 secretion induced in the presence of Th17-polarizing cytokines was significantly suppressed by siRNA IL-1RI (Figure 4B). IL-17A, IL-17F, IL-21, and IL-22 are significantly induced in MS patients compared to HCs at baseline, and upon stimulation with Th17-polarizing cytokines (Figures 4A,B), confirming preferential IL-1R-induced Th17 cell differentiation in RR MS.

### IL-1RI Induction of Th17 Cell Differentiation Is IRF4-Dependent

**Figure 5:**
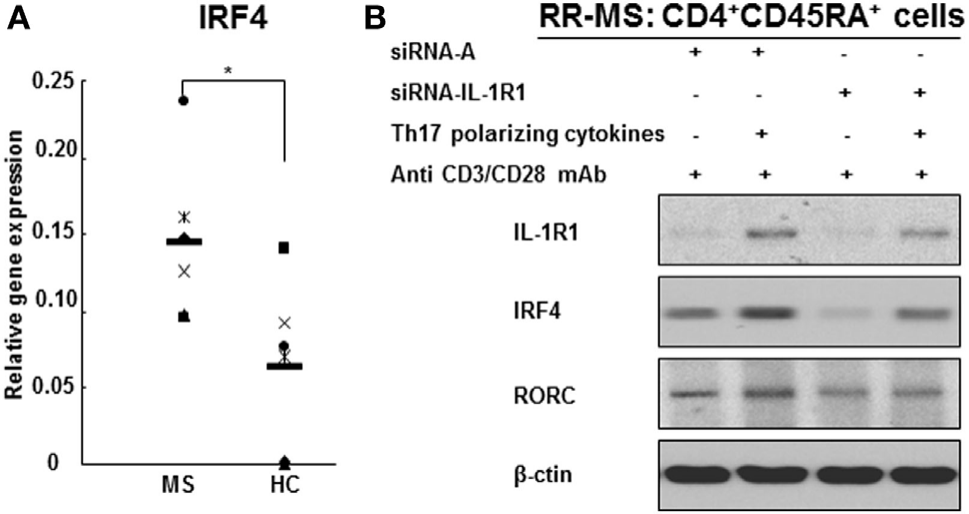
**IL-1R1 and IRF4 are required for human Th17 cell differentiation**. **(A)** IRF4 gene expression in the CD4^+^ T cell from RR MS patients is significantly increased in comparison to HCs. CD4^+^ T cells derived from six RR MS patients and six HCs were separated using magnetic beads, and the total RNA was extracted. The gene expression of IRF4 and 18S was measured by RT-PCR. The results are expressed as relative gene expression normalized for 18S mRNA expression. Statistical analysis was performed using *t*-tests. *p < 0.05. **(B)** IL-1R1 gene silencing downregulates IRF4 and RORc protein expression in CD4^+^CD45RA^+^ cells from RR MS patients and inhibits Th17 cell differentiation.

**Figure 6:**
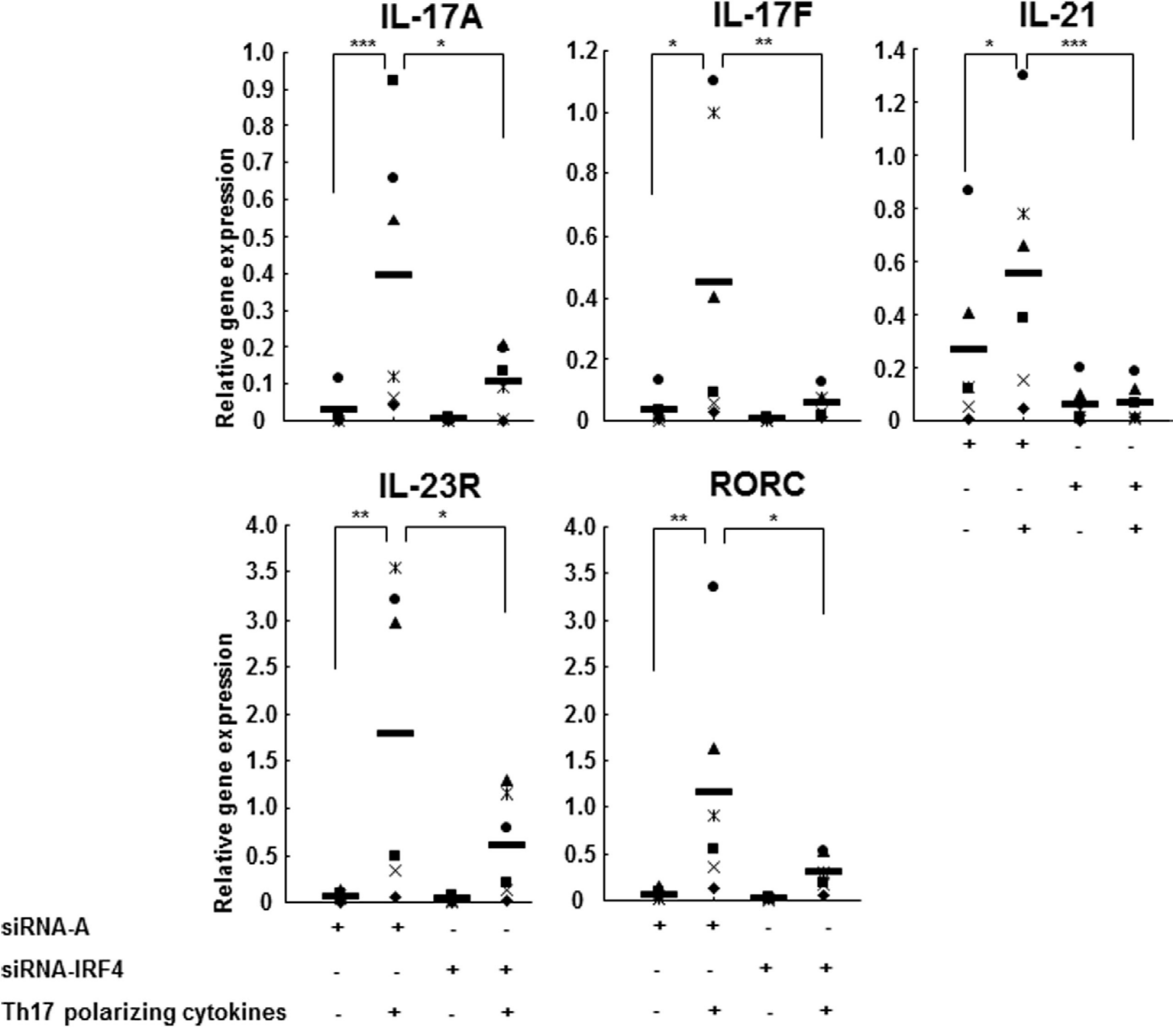
**IL-1R1 positively regulates human Th17 cell differentiation in an IRF4-dependent manner**. CD4^+^CD45RA^+^ cells from six RR MS patients transfected with a control siRNA A or siRNA IRF4 were stimulated with plate-immobilized anti-CD3 and anti-CD28 mAb and cultured in serum-free medium in the absence or presence of Th17-polarizing cytokines. After 72 h, the total RNA was extracted. The gene expression was measured by RT-PCR. The results are expressed as relative gene expression normalized for 18S mRNA expression. Statistical analysis was performed using ANOVA. *p < 0.05, **p < 0.01, ***p < 0.001.

Interleukin regulatory factor 4 gene expression in CD4^+^ T cells derived from RR MS patients was significantly increased in comparison to those from HCs (Figure 5A). Western blotting studies of the naive CD4^+^CD45RA^+^ cells transfected with siRNA IL-1RI demonstrated a significant inhibition of IL-1RI protein expression (85% suppression), which was associated with a decreased expression of IRF4 and RORc in both HCs and RR MS patients (Figure 5B). These results indicate that IL-1RI signaling induces IRF4 and RORc expression in human naive CD4^+^ cells. In order to directly examine the role of IL-1RI and IRF4 signaling in human Th17 cell differentiation, the IRF4 gene expression was silenced using a siRNA approach. The naive CD4^+^CD45RA^+^ cells from RR MS patients, which were transfected with siRNA IRF4, exhibited significant inhibition of the induction of IL-1RI, IRF4, RORc, IL-17A, IL-17F, IL-21, and IL-23R gene expression (Figure 6).

## Discussion

This study has demonstrated that IL-1RI expression is significantly higher in both the naive and memory CD4^+^ T cells derived from RR MS patients in comparison to those from HCs. *In vitro*-differentiated Th17 cells express higher levels of IL-RI than Th1- or Th2-differentiated cells. Finally, the siRNA silencing of IL-1RI in naive CD4^+^ cells inhibited *in vitro* Th17 cell differentiation, by inhibiting IRF4 and RORc, as well as IL-17A, IL-17F, IL-21, IL-22, and IL-23R gene expression. Cytokine secretion measurements detected a significantly decreased IL-17A, IL-17F, and IL-21 secretion by the siRNA IL-1R1-transfected naive *in vitro* polarized Th17 cells, indicating that IL-1R1 signaling induces Th17 cell differentiation. Our study has identified that increased IL-1R1 expression in multiple CD4^+^ cell subsets may reflect this pathway’s activation in patients with RR MS, leading to an increased Th17 cell expansion and autoimmune response.

Interleukin regulatory factor 4 is a transcription factor that serves as a key regulator of both Th2 and Th17 differentiation (24, 25). Our previously published results identified IRF4 as a key transcription factor for the human Th17 cell differentiation using both IRF4 gene expression silencing and overexpression (26). IRF4 induces Th2 differentiation *via* upregulation of the Th2 transcription factor GATA-3 and interaction with the transcription factor NFAT (27–29). IRF4-deficient T helper cells had less expression of RORγt and more expression of Foxp3 and did not differentiate to Th17 cells. IRF4-deficient mice are protected from EAE (24). The transcription factor RORγt plays a crucial role in the development of Th17 cells (30). IRF4 deficiency is associated with a decrease in IL-6-induced RORγt expression that impedes Th17 differentiation (24). The published observations have shown that IRF4 controls experimental colitis in mice *via* T cell-derived IL-6 (31), and that IL-6 suppresses TGFβ-induced development of regulatory T cells and Foxp3 expression (4, 32). In normal Th17 cells, IL-6-mediated downregulation of Foxp3 is needed to counteract the upregulation of Foxp3 by TGFβ (33). IRF4 can interact with NFATc1 and NFATc2 in T helper cells (27, 29) and with the transcription factors PU.1 and STAT6 in B cells (34, 35). In addition, IRF4-binding protein (IBP) inhibits IL-17 and IL-21 production by controlling IRF4 function, which directly binds to the IL-17 and IL-21 promoters and induces their transcriptional activation (36). More recently, it has been reported that IRF4 is also crucial for the development and function of an IL-9-producing CD4^+^ T cell subset designated Th9. The study has reported that IL-9 production in human CD4^+^ T cells is induced by the upregulation of IRF4 (37).

IL-1RI signaling is a critical step in human Th17 cell differentiation, leading to the induction of IRF4 and RORc. In our study, the expressions of IL-1RI, IRF4, and RORc increased upon stimulation with Th17-polarizing cytokines in CD4^+^CD45RA^+^ cells derived from RR MS patients and HCs, indicating that IRF4 and RORc are Th17 cell lineage transcription factors. IL-1RI gene silencing by siRNA in naive CD4^+^CD45RA^+^ cells leads to a significant inhibition of IL-1RI protein expression, as well as IRF4 and RORc, in both HCs and RR MS patients in a Th17-polarizing condition. When we knocked down the IRF4 gene expression in naive CD4^+^ cells using siRNA IRF4, Th17-polarizing condition failed to induce Th17 cell differentiation and the expression of RORc was inhibited, indicating that RORc signals downstream of IRF4. Activated IL-1RI induces IRF4 expression, which causes the activation and induction of RORc and IL-17. Our results indicate that IL-1RI signaling may induce IRF4 and RORc protein expression during human Th17 cell differentiation, consistent with the results by Chung et al. who reported that IL-1RI-deficient mouse T cells failed to express IRF4 and RORc (12).

The IL-1R1 signaling pathway has received a renewed attention due to its recently reported induction of transcription factor Bhlhe40 associated with Th17 cell encephalitogenic phenotype and GM-CSF secretion (38). Furthermore, the role of IL-1 signaling in the generation of autoimmune responses was most recently documented by the production of IL-1 by neutrophils and monocytes as they migrate to the CNS and induce IL-1R signaling in endothelial cells, which trigger the release of proinflammatory cytokines and chemokines that enhance neutrophil and monocyte recruitment and further T cell recruitment to the CNS (39). Its complex regulation by multiple naturally occurring inhibitors, including recombinant IL-R antagonist opens new possibilities in the treatment of autoimmune diseases and particularly of MS (40, 41). New strategies for the selective targeting of only some of the effector mechanisms of the IL-1RI signaling pathway using selective reversible non-competitive small peptide antagonist of IL-1RI hold promise as safe and effective treatments for autoimmune diseases (42).

## Author Contributions

YS performed the experiments and wrote the paper; SM-P designed the study and wrote the paper.

## Conflict of Interest Statement

The authors declare that the research was conducted in the absence of any commercial or financial relationships that could be construed as a potential conflict of interest.

## Abbreviations

EAE, experimental autoimmune encephalomyelitis; HCs, healthy controls; IL-1R, interleukin-1 receptor; IRF, interferon regulatory factor; PBMCs, peripheral blood mononuclear cells; RR MS, relapsing-remitting multiple sclerosis; SNs, supernatants, TCR, T cell receptor.
